# Poly[di-μ_2_-aqua-μ_4_-chlorido-μ_4_-(2-mercaptopyrimidine-4,6-diolato-κ^4^ *O*:*O*:*O*′:*O*′)-disodium(I)]

**DOI:** 10.1107/S1600536810045836

**Published:** 2010-11-13

**Authors:** Bao Li, Wen Li, Ling Ye, Guang-Feng Hou, Li-Xin Wu

**Affiliations:** a State Key Laboratory of Supramolecular Structure and Materials, College of Chemistry, Jilin University, Changchun 130012, People’s Republic of China

## Abstract

In the title coordination polymer, [Na_2_(C_4_H_3_N_2_O_2_S)Cl(H_2_O)_2_]_*n*_, the Na^I^ ion lies on a twofold rotation axis and the chloride anion on an inversion center. The Na^I^ ion is six-coordinated by two O atoms from two zwitterionic 2-mercaptopyrimidine-4,6-diolate ligands (*mm*2 symmetry), two water O atoms (*m* symmetry) and two Cl atoms in a distorted octa­hedral geometry. Adjacent Na^I^ ions are bridged by an olate group, a water mol­ecule and a chloride anion into a three-dimensional network. The crystal structure is further stabil­ized by N—H⋯Cl, O—H⋯O and O—H⋯S hydrogen bonds.

## Related literature

For organic–inorganic hybrid compounds with 2-mercaptopyrimidine-4,6-diol derivatives, see: Carballo *et al.* (1996 ▶).
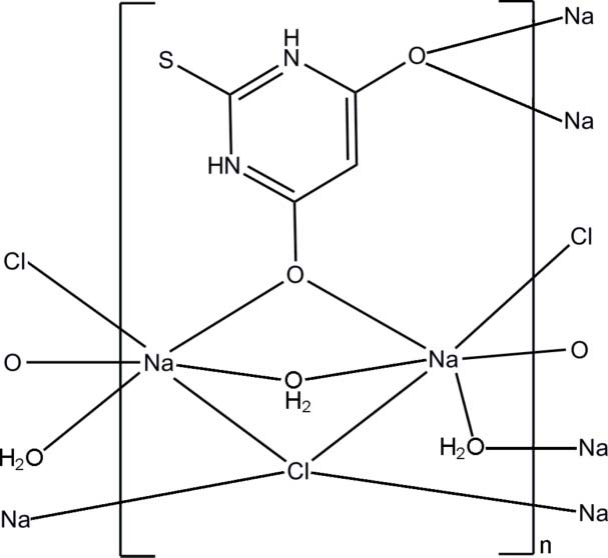


## Experimental

### 

#### Crystal data

[Na_2_(C_4_H_3_N_2_O_2_S)Cl(H_2_O)_2_]
*M* *_r_* = 260.61
Orthorhombic, 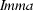

*a* = 16.815 (3) Å
*b* = 6.5938 (13) Å
*c* = 8.8587 (18) Å
*V* = 982.2 (3) Å^3^
*Z* = 4
Mo *K*α radiation
μ = 0.68 mm^−1^
*T* = 290 K
0.12 × 0.11 × 0.09 mm

#### Data collection

Rigaku R-AXIS RAPID diffractometer
Absorption correction: multi-scan (*ABSCOR*; Higashi, 1995 ▶) *T*_min_ = 0.923, *T*_max_ = 0.942
4752 measured reflections
633 independent reflections
581 reflections with *I* > 2σ(*I*)
*R*_int_ = 0.017

#### Refinement

*R*[*F* ^2^ > 2σ(*F* ^2^)] = 0.024
*wR*(*F* ^2^) = 0.062
*S* = 1.09
633 reflections
46 parameters
H-atom parameters constrained
Δρ_max_ = 0.26 e Å^−3^
Δρ_min_ = −0.30 e Å^−3^

### 

Data collection: *RAPID-AUTO* (Rigaku, 1998 ▶); cell refinement: *RAPID-AUTO*; data reduction: *CrystalStructure* (Rigaku/MSC, 2002 ▶); program(s) used to solve structure: *SHELXS97* (Sheldrick, 2008 ▶); program(s) used to refine structure: *SHELXL97* (Sheldrick, 2008 ▶); molecular graphics: *SHELXTL* (Sheldrick, 2008 ▶); software used to prepare material for publication: *PLATON* (Spek, 2009 ▶).

## Supplementary Material

Crystal structure: contains datablocks global, I. DOI: 10.1107/S1600536810045836/hy2376sup1.cif

Structure factors: contains datablocks I. DOI: 10.1107/S1600536810045836/hy2376Isup2.hkl

Additional supplementary materials:  crystallographic information; 3D view; checkCIF report

## Figures and Tables

**Table 1:** Hydrogen-bond geometry (Å, °)

*D*—H⋯*A*	*D*—H	H⋯*A*	*D*⋯*A*	*D*—H⋯*A*
N1—H1⋯Cl1	0.86	2.41	3.2457 (15)	165
O2—H2*A*⋯O1^i^	0.89	1.94	2.8164 (19)	169
O2—H2*B*⋯S1^ii^	0.87	2.49	3.3545 (15)	176

Symmetry codes: (i) 
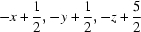
; (ii) 
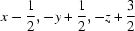
.

**Fig. 1.**
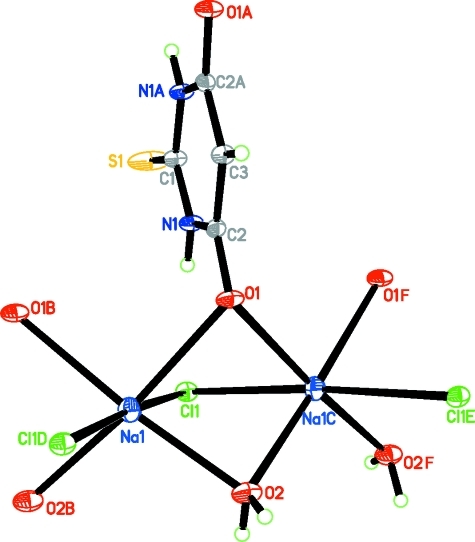
The asymmetric unit of the title compound, with symmetry-related atoms to complete the ligand and Na coordination. Displacement ellipsoids are drawn at the 30% probability level. [Symmetry codes: (A): 1-x, 1/2-y, z; (B): x, 1-y, 2-z; (C): x, -1/2+y, 2-z; (D): 1/2-x, 1-y, 1/2+z; (E): 1/2-x, -y, 1/2+z; (F) x, -y, 2-z.]

## supplementary crystallographic information

### Comment

2-Mercaptopyrimidine-4,6-diol and its derivatives have been used widely to build organic-inorganic hybrids due to their diverse properties (Carballo *et al.* 1996). During the course of on-going crystal engineering studies on nickel salts, we obtained two types of crystals different in color, green and colorless. We herein report the crystal structure of the colorless one, the title compound.

The coordination environment around Na^I^ ion is shown in Fig. 1, with atom numbering scheme. The Na^I^ ion is six-coordinated in a distorted octahedral geometry by two O atoms from two 2-mercaptopyrimidine-4,6-diolate ligands, two water O atoms and two Cl atoms. The 2-mercaptopyrimidine-4,6-diolate anion serving as a bridging ligand coordinates to four Na^I^ ions. The crystal structure is stabilized by N—H···Cl, O—H···O and O—H···S hydrogen bonds (Table 1).

### Experimental

2-Mercaptopyrimidine-4,6-diol (1.44 g, 10 mmol) and NiCl_2_.6H2O (3.28 g, 10 mmol) were dissolved in hot water (20 ml) and the pH value was adjusted to about 5 by using dilute NaOH solution with stirring. The mixture was heated for one hour and then cooled to room temperature. The precipitate was washed by dilute HCl and the filtrate was allowed to evaporate at room temperature for two weeks, generating two types of block crystals, one was colorless and the other was green.

### Refinement

C- and N-bound H atoms were positioned geometrically (C—H = 0.93, N—H = 0.86 Å) and refined as riding atom, with *U*_iso_(H) = 1.2*U*_eq_(C,N). Water H atoms were located in a difference Fourier map and refined as riding, with *U*_iso_(H) = 1.5*U*_eq_(O).

### Crystal data

**Table:**

[Na_2_(C_4_H_3_N_2_O_2_S)Cl(H_2_O)_2_]	*F*(000) = 528
*M**_r_* = 260.61	*D*_x_ = 1.762 Mg m^−^^3^
Orthorhombic, *I**m**m**a*	Mo *K*α radiation, λ = 0.71073 Å
Hall symbol: -I 2b 2	Cell parameters from 4272 reflections
*a* = 16.815 (3) Å	θ = 3.3–27.4°
*b* = 6.5938 (13) Å	µ = 0.68 mm^−^^1^
*c* = 8.8587 (18) Å	*T* = 290 K
*V* = 982.2 (3) Å^3^	Block, colorless
*Z* = 4	0.12 × 0.11 × 0.09 mm

### Data collection

**Table:**

Rigaku R-AXIS RAPID diffractometer	633 independent reflections
Radiation source: rotation anode	581 reflections with *I* > 2σ(*I*)
graphite	*R*_int_ = 0.017
ω scans	θ_max_ = 27.4°, θ_min_ = 3.9°
Absorption correction: multi-scan (*ABSCOR*; Higashi, 1995)	*h* = −21→19
*T*_min_ = 0.923, *T*_max_ = 0.942	*k* = −8→8
4752 measured reflections	*l* = −11→11

### Refinement

**Table:**

Refinement on *F*^2^	Primary atom site location: structure-invariant direct methods
Least-squares matrix: full	Secondary atom site location: difference Fourier map
*R*[*F*^2^ > 2σ(*F*^2^)] = 0.024	Hydrogen site location: inferred from neighbouring sites
*wR*(*F*^2^) = 0.062	H-atom parameters constrained
*S* = 1.09	*w* = 1/[σ^2^(*F*_o_^2^) + (0.0282*P*)^2^ + 1.0461*P*] where *P* = (*F*_o_^2^ + 2*F*_c_^2^)/3
633 reflections	(Δ/σ)_max_ = 0.002
46 parameters	Δρ_max_ = 0.26 e Å^−^^3^
0 restraints	Δρ_min_ = −0.30 e Å^−^^3^

### Fractional atomic coordinates and isotropic or equivalent isotropic displacement parameters (Å^2^)

**Table:**

	*x*	*y*	*z*	*U*_iso_*/*U*_eq_	
C1	0.5000	0.2500	0.7898 (3)	0.0222 (5)	
C2	0.42785 (10)	0.2500	1.02887 (18)	0.0174 (3)	
C3	0.5000	0.2500	1.1064 (3)	0.0199 (5)	
H3	0.5000	0.2500	1.2113	0.024*	
Cl1	0.2500	0.2500	0.7500	0.02138 (17)	
N1	0.43218 (8)	0.2500	0.87105 (16)	0.0204 (3)	
H1	0.3881	0.2500	0.8220	0.025*	
Na1	0.25570 (4)	0.5000	1.0000	0.0256 (2)	
O1	0.35874 (7)	0.2500	1.08459 (14)	0.0242 (3)	
O2	0.16839 (8)	0.2500	1.10168 (15)	0.0294 (3)	
H2A	0.1533	0.2500	1.1978	0.044*	
H2B	0.1232	0.2500	1.0538	0.044*	
S1	0.5000	0.2500	0.60136 (8)	0.0531 (3)	

### Atomic displacement parameters (Å^2^)

**Table:**

	*U*^11^	*U*^22^	*U*^33^	*U*^12^	*U*^13^	*U*^23^
C1	0.0155 (11)	0.0313 (13)	0.0197 (11)	0.000	0.000	0.000
C2	0.0158 (7)	0.0182 (7)	0.0182 (8)	0.000	0.0019 (6)	0.000
C3	0.0178 (11)	0.0262 (12)	0.0158 (10)	0.000	0.000	0.000
Cl1	0.0201 (3)	0.0286 (3)	0.0154 (3)	0.000	−0.0028 (2)	0.000
N1	0.0127 (7)	0.0314 (8)	0.0172 (7)	0.000	−0.0013 (5)	0.000
Na1	0.0288 (4)	0.0236 (4)	0.0245 (4)	0.000	0.000	−0.0031 (3)
O1	0.0141 (6)	0.0382 (7)	0.0204 (6)	0.000	0.0030 (5)	0.000
O2	0.0206 (6)	0.0449 (8)	0.0225 (6)	0.000	0.0013 (5)	0.000
S1	0.0199 (3)	0.1243 (10)	0.0150 (3)	0.000	0.000	0.000

### Geometric parameters (Å, °)

**Table:**

C1—N1	1.3484 (19)	N1—H1	0.8600
C1—S1	1.670 (3)	Na1—O2	2.3842 (11)
C2—O1	1.263 (2)	Na1—O1	2.5062 (11)
C2—C3	1.394 (2)	Na1—Cl1^i^	2.7625 (4)
C2—N1	1.400 (2)	Na1—Na1^ii^	3.2969 (6)
C3—H3	0.9300	O2—H2A	0.8885
Cl1—Na1	2.7625 (4)	O2—H2B	0.8701
			
N1^iii^—C1—N1	115.5 (2)	O2—Na1—Cl1^i^	95.07 (3)
N1^iii^—C1—S1	122.25 (11)	O2^ii^—Na1—Cl1^i^	82.47 (3)
N1—C1—S1	122.25 (11)	O1^ii^—Na1—Cl1^i^	82.60 (3)
O1—C2—C3	127.48 (16)	O1—Na1—Cl1^i^	100.18 (3)
O1—C2—N1	115.99 (15)	Cl1—Na1—Cl1^i^	176.03 (3)
C3—C2—N1	116.53 (15)	O2—Na1—Na1^ii^	133.74 (3)
C2^iii^—C3—C2	121.0 (2)	O2^ii^—Na1—Na1^ii^	46.26 (3)
C2^iii^—C3—H3	119.5	O1^ii^—Na1—Na1^ii^	48.87 (2)
C2—C3—H3	119.5	O1—Na1—Na1^ii^	131.13 (2)
Na1—Cl1—Na1^iv^	180.0	Cl1—Na1—Na1^ii^	126.636 (8)
Na1—Cl1—Na1^v^	73.272 (15)	Cl1^i^—Na1—Na1^ii^	53.364 (8)
Na1^iv^—Cl1—Na1^v^	106.728 (15)	O2—Na1—Na1^v^	46.26 (3)
Na1—Cl1—Na1^vi^	106.728 (15)	O2^ii^—Na1—Na1^v^	133.74 (3)
Na1^iv^—Cl1—Na1^vi^	73.272 (16)	O1^ii^—Na1—Na1^v^	131.13 (2)
Na1^v^—Cl1—Na1^vi^	180.0	O1—Na1—Na1^v^	48.87 (2)
C1—N1—C2	125.23 (16)	Cl1—Na1—Na1^v^	53.364 (8)
C1—N1—H1	117.4	Cl1^i^—Na1—Na1^v^	126.636 (8)
C2—N1—H1	117.4	Na1^ii^—Na1—Na1^v^	180.00 (5)
O2—Na1—O2^ii^	103.98 (6)	C2—O1—Na1^v^	121.29 (7)
O2—Na1—O1^ii^	173.45 (5)	C2—O1—Na1	121.29 (7)
O2^ii^—Na1—O1^ii^	81.84 (4)	Na1^v^—O1—Na1	82.26 (4)
O2—Na1—O1	81.84 (4)	Na1—O2—Na1^v^	87.48 (5)
O2^ii^—Na1—O1	173.45 (5)	Na1—O2—H2A	122.5
O1^ii^—Na1—O1	92.53 (5)	Na1^v^—O2—H2A	122.5
O2—Na1—Cl1	82.47 (3)	Na1—O2—H2B	110.7
O2^ii^—Na1—Cl1	95.07 (3)	Na1^v^—O2—H2B	110.7
O1^ii^—Na1—Cl1	100.18 (3)	H2A—O2—H2B	102.6
O1—Na1—Cl1	82.60 (3)		

Symmetry codes: (i) −*x*+1/2, −*y*+1, *z*+1/2; (ii) *x*, *y*+1/2, −*z*+2; (iii) −*x*+1, −*y*+1/2, *z*; (iv) −*x*+1/2, −*y*+1/2, −*z*+3/2; (v) *x*, *y*−1/2, −*z*+2; (vi) −*x*+1/2, −*y*+1, *z*−1/2.

### Hydrogen-bond geometry (Å, °)

**Table:**

*D*—H···*A*	*D*—H	H···*A*	*D*···*A*	*D*—H···*A*
N1—H1···Cl1	0.86	2.41	3.2457 (15)	165
O2—H2A···O1^vii^	0.89	1.94	2.8164 (19)	169
O2—H2B···S1^viii^	0.87	2.49	3.3545 (15)	176

Symmetry codes: (vii) −*x*+1/2, −*y*+1/2, −*z*+5/2; (viii) *x*−1/2, −*y*+1/2, −*z*+3/2.
